# Synergistic Enhancement of Freeze–Thaw Durability and Structural Integrity in Silty Clay Through Combined Microbial Carbonate Precipitation and Anionic Polyacrylamide Modification

**DOI:** 10.3390/ma19132702

**Published:** 2026-06-23

**Authors:** Hongfeng Li, Zijie Wei, Yanfang Tong, Dahong Yang, Guang-Zhu Zhang

**Affiliations:** 1 School of Civil Engineering and Transportation, Northeast Forestry University, Harbin 150040, China; lihongfeng@nefu.edu.cn (H.L.); weizijie0828@163.com (Z.W.); 2 Heilongjiang Longjian Road and Bridge Third Engineering Co., Ltd., Harbin 150009, China; wh892058724@163.com; 3 Heilongjiang Longjian Road and Bridge Sixth Engineering Co., Ltd., Harbin 150076, China; 15331886933@189.cn

**Keywords:** microbially induced carbonate precipitation, anionic polyacrylamide, freeze–thaw durability, silty clay, pore structure reconstruction

## Abstract

**Highlights:**

- A sustainable MICP–APAM composite approach improved silty clay strength and freeze–thaw durability.
- OD600 ≈ 1.0 balanced nucleation and diffusion, forming a uniform cluster–film network.
- APAM bridging and MICP mineralization enhanced pore refinement, bonding, and compactness.
- Multi-evidence analyses confirmed fine, homogeneous CaCO_3_ and strong polymer–mineral adhesion.

**Abstract:**

Seasonal freeze–thaw cycling progressively rearranges pores and propagates microcracks in silty clay, reducing the reliability of cold-region earthworks. This study evaluated a bio–polymer stabilization strategy combining microbially induced carbonate precipitation (MICP) with anionic polyacrylamide (APAM) to improve mechanical performance and freeze–thaw durability. Six groups were prepared at identical moisture and compaction conditions: water, APAM, and four MICP–APAM groups with bacterial optical densities (OD600) of 0.8, 1.0, 1.2, and 1.4. Unconfined compressive strength, unconsolidated-undrained triaxial compression, ultrasonic pulse velocity, and SEM, TG/DTG, XRD, and FTIR analyses were conducted before and after freeze–thaw cycling. The M1.0-APAM group showed the best overall performance, with UCS values of 1.35 MPa before cycling and 0.89 MPa after nine cycles, together with high shear resistance and ultrasonic velocity. Lower bacterial concentration provided insufficient cementation, whereas higher concentrations promoted non-uniform carbonate deposition, pore heterogeneity, and local stress concentration. Microstructural evidence indicated that OD600 ≈ 1.0 produced a relatively homogeneous network of fine carbonate clusters and polymer-associated films, with calcite formation supported by TG/DTG and XRD. The results show that MICP–APAM treatment enhances silty clay primarily through coordinated mineralization uniformity, pore refinement, and polymer bridging, providing a sustainable stabilization option for seasonally frozen soils.

## 1. Introduction

Seasonal freeze–thaw cycles cause repeated pore rearrangement and microcrack propagation in soils under coupled hydrothermal fluctuations, leading to progressive deterioration in strength and stiffness and a marked increase in erosion susceptibility. The black soil region of Northeast China is a typical representative of such environments [1,2]. In this region, the winter temperature remains around −20 °C and may drop to −40 °C under extreme conditions [3,4]. Black soils are characterized by a thick humic horizon, high organic matter content, and strong water retention capacity [5,6]. Under the combined influence of gravity-driven runoff and freeze–thaw alternation, both natural gully erosion and secondary channel formation accelerate, further undermining the structural integrity of the soil [7,8,9,10]. In infrastructure scenarios such as roadbeds and slopes, these degradation mechanisms result in cumulative reductions in load-bearing capacity and long-term durability, highlighting the need to identify controlling factors and develop sustainable soil stabilization and recovery strategies.

Existing soil improvement methods can be broadly classified into chemical, physical, and biological approaches. Chemical stabilization using cement [11], lime [12], or fly ash [13] can significantly enhance soil strength and stiffness [14], but it may introduce environmental burdens and structural disturbances, including heavy-metal migration and soil–water pollution [15]. Physical modification methods, such as incorporating plastic waste [16], rubber particles [17], or polymeric materials [18], can alter the soil skeleton and pore structure. Although some plastic materials have been reported to lower the freezing point of pore water [19], their poor degradability causes long-term accumulation in soil and water environments, posing ecological risks [20]. Rubber can improve damping and energy dissipation but often reduces the initial shear modulus, requiring a balance between vibration isolation and load capacity, and tire-derived particles are now recognized as an important source of microplastics and toxic additives in urban runoff [21,22]. Organic polymers have been widely used in soil stabilization due to their biocompatibility, low toxicity, and flocculation capacity [23,24,25].

Biological stabilization techniques mainly include microbially induced calcium carbonate precipitation (MICP), enzyme-induced carbonate precipitation (EICP), and microbially induced phosphate precipitation (MIPP). MICP can substantially improve strength and impermeability but is sensitive to curing and environmental conditions [26]. EICP offers simpler management and improved permeability [27], but enzyme cost and stability limit its scalability [28]. MIPP provides better chemical stability and can immobilize heavy metals through co-precipitation [29], yet the reaction is relatively slow and sensitive to pH and ionic strength [30] Considering the dual demands of mechanical performance and environmental compatibility, hybrid stabilization approaches that integrate physical and biological mechanisms have greater engineering potential and sustainability.

In MICP-related applications, typical bacterial strains include *Paracoccus denitrificans*, *Bacillus subtilis*, and *Sporosarcina pasteurii*. *P. denitrificans* induces CaCO_3_ precipitation through denitrification and is suitable for medium-permeability sands, although its reaction is slow and nitrate-dependent [31,32]. *B. subtilis* produces extracellular polymeric substances that enhance cohesion, erosion resistance, and ductility, commonly used for the surface reinforcement of slopes and fine-grained soils [33]. *S. pasteurii* exhibits high urease activity and environmental tolerance, making it well-suited for media ranging from coarse sand to silty clay and showing strong adaptability for engineering use [34].

Among the physical stabilizers, polymeric materials not only enhance strength, impermeability, and frost resistance, but also regulate the moisture and ionic microenvironment, buffer pH fluctuations, mitigate shear disturbance, and provide nucleation and bonding sites that promote microbial carbonate deposition [35]. Various polymers such as polyacrylamide (PAM), polyacrylates, polyvinyl alcohol (PVA), polyvinyl acetate (PVAc), and polyurethanes have been evaluated as potential stabilizers [36]. Among them, PAM has long been used in agricultural irrigation and soil conditioning due to its environmental compatibility and stability, providing a foundation for polymer-assisted MICP applications [37,38].

As a green stabilization approach, the coupling of MICP with polymers such as anionic polyacrylamide (APAM) is moving from mechanistic research toward engineering application. In seasonally frozen environments, APAM serves as a representative polymer for synergistic microbial–polymer stabilization. The carboxylate groups (–COO^−^) in APAM chains can strongly adsorb and complex with Ca^2+^, generating interparticle bridging that refines pore structure, increases pore-water viscosity, and reduces the supply of unfrozen water to the freezing front, thereby improving freeze–thaw stability [39]. Meanwhile, the polymer network provides a moisture-retaining, diffusion-regulating, and mechanically buffered microenvironment that sustains microbial activity and enzymatic mineralization efficiency under variable temperature and humidity. Molecular simulations and parameterized experiments have demonstrated significant interfacial adhesion and ionic bridging between PAM and CaCO_3_, establishing its material basis as a nucleation template and crystal-anchoring medium [40]. Hence, under reduced chemical input and environmental impact, the APAM–MICP synergy is expected to enhance both the strength and durability of soils subjected to freeze–thaw cycles.

Although MICP and polymer stabilization have been widely investigated, the coupled role of APAM and bacterial concentration in controlling carbonate spatial distribution, pore-network reconstruction, and freeze–thaw durability of silty clay remains insufficiently defined. In particular, the relationship among OD600-controlled bacterial level, APAM chain bridging, mineralization uniformity, and post-freeze–thaw mechanical stability has not been systematically clarified. The present study addresses this gap by integrating UCS, UU triaxial testing, UPV, SEM, TG/DTG, XRD, and FTIR evidence to identify the optimal MICP–APAM balance and to establish a multi-scale mechanism for cold-region silty clay stabilization.

## 2. Materials and Methods

### 2.1. Materials

#### 2.1.1. Silty Clay

**Figure 1:**
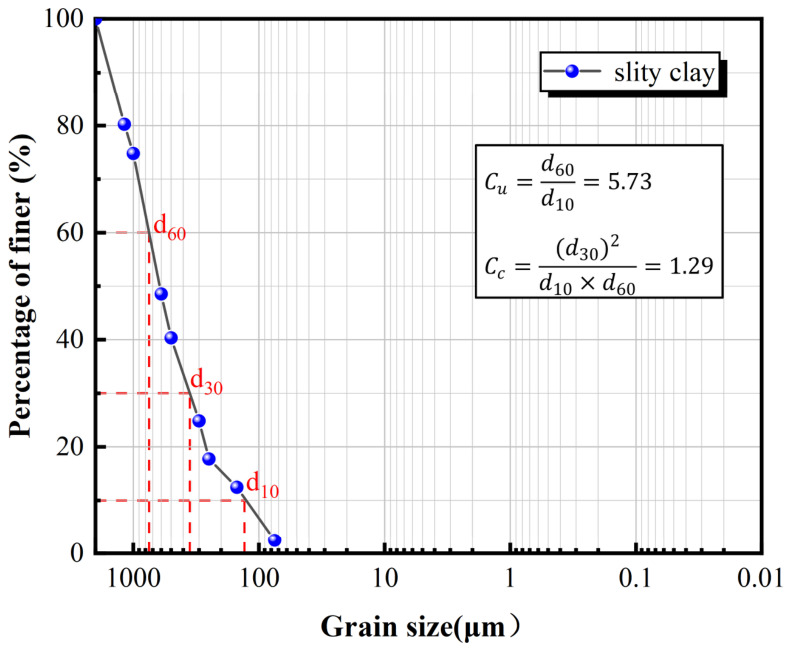
Particle-size distribution curve of the silty clay.

**Figure 2:**
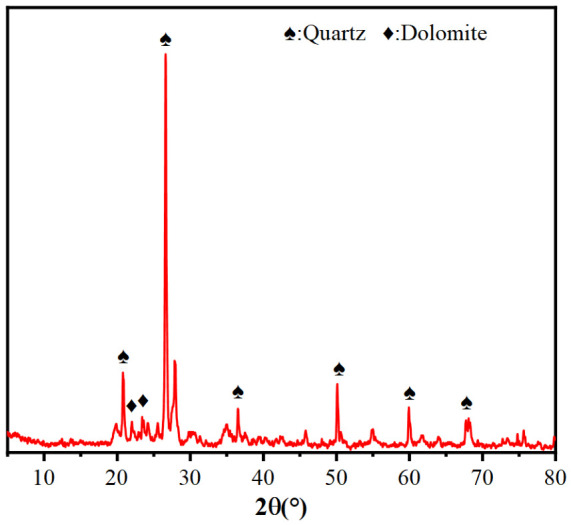
XRD pattern of the silty clay.

Silty clay was sampled from the black soil region of Northeast China. Vegetation roots and visible debris were removed, the soil was air-dried, gently disaggregated, and sieved to obtain the grading required for laboratory testing, with a lower particle-size cutoff of 0.075 mm and passage through a 2.0 mm sieve. Basic physical properties were determined in accordance with GB/T 50123-2019 [41], including water content determination by the oven-drying method, liquid and plastic limit tests, and light compaction testing. Particle-size analysis indicates a fines-dominated matrix with approximately 30% clay and coarser silt as the secondary fraction. Representative indices are summarized in Table 1. The particle-size distribution curve is shown in Figure 1. X-ray diffraction (XRD) results are shown in Figure 2. The dominant crystalline minerals identified in the silty clay are dolomite and quartz.

#### 2.1.2. APAM Solution

APAM (Product No. 30503770, “Polyacrylamide, MW ≈ 10^7^, anionic type”, CAS No. 9003-05-8) was supplied by Sinopharm Chemical Reagent Co., Ltd. (Shanghai, China). APAM is a linear, anionically modified polymer whose repeating unit is derived from acrylamide [–CH_2_–CH(CONH_2_)–]_n_. At room temperature, it appears as white to light-yellow granules or powder, soluble in deionized water to form a translucent viscous solution. The carboxylate and amide functional groups along the polymer chain can interact with soil particle surfaces through electrostatic attraction, ionic bridging, and hydrogen bonding, forming an interconnected spatial network that stabilizes soil structure and regulates the interparticle bonding framework. This polymeric network also provides a microenvironment favorable for ion enrichment, water retention, and diffusion control during the MICP process. To prepare the stock solution, the APAM powder was slowly sprinkled into vigorously stirred deionized water and mixed for 1–2 h until complete hydration and dispersion were achieved, followed by degassing at rest. The dry powder was stored in sealed, dry, and light-proof conditions at 15–25 °C. The stock solution was stored at 4 °C in the dark and used within a short period. Before use, the stock solution was gently stirred to ensure homogeneity, avoiding strong shear that could degrade the molecular chains and reduce molecular weight.

#### 2.1.3. *Sporosarcina pasteurii*

The ureolytic bacterium *Sporosarcina pasteurii* (formerly *Bacillus pasteurii*, strain ATCC 11859) was employed as the MICP-inducing microorganism [42]. It is a Gram-positive, aerobic, endospore-forming bacterium with high urease activity capable of hydrolyzing urea to generate CO_3_^2−^, which subsequently reacts with Ca^2+^ to precipitate CaCO_3_. The strain, classified as biosafety level BSL-1, was obtained from the Guangdong Microbial Culture Collection Center (China). Bacterial cultivation was conducted at 30 ± 2 °C with shaking at 150–200 rpm, and the initial pH was maintained between 7.5 and 9.0. The bacterial concentration was quantified using optical density at 600 nm (OD_600_), with target values of 0.8, 1.0, 1.2, and 1.4. Samples were collected at the late exponential or stationary growth phase and stored short-term on agar plates at 4 °C. All inoculation and transfer operations were performed in a laminar-flow cabinet to maintain aseptic conditions and reproducibility.

#### 2.1.4. MICP Culture Medium and Chemical Components

The cultivation and ureolysis-based MICP procedures for *S. pasteurii* were based on an established protocol [43], with the specific formulations and operating conditions used in the present study described below. To ensure experimental consistency, all culture media and chemical reagents were prepared from the same batches under identical conditions. Deionized water served as the solvent, and the standard culture composition (per liter) consisted of 6.00 g beef extract, 2.13 g NaHCO_3_, 13.33 g NH_4_Cl, and 111.0 g CaCl_2_, with the initial pH adjusted to 7.0. This medium was used for both bacterial proliferation and under sterile conditions, as the cementation solution for MICP treatment.

(1) Reagents and their roles

Anhydrous CaCl_2_ (analytical grade; Tianjin Yongda Chemical Reagent Co., Ltd., Tianjin, China; HG/T 5349–2018 [44]) was used as the calcium source, with CaCl_2_ content ≥ 96%. Sodium bicarbonate (analytical grade; Tianjin Yongda Chemical Reagent Co., Ltd., Tianjin, China; GB/T 640–1997 [45]) served as the inorganic carbon source and buffer, with purity ≥99.5%. Ammonium chloride (analytical grade; Tianjin Kaitong Chemical Reagent Co., Ltd., Tianjin, China; GB/T 658–2006 [46]) provided nitrogen and ionic strength regulation, exhibiting a pH of 4.5–5.5 for a 50 g·L^−1^ aqueous solution at 25 °C. Beef extract (Beijing AoBoXing Biotechnology Co., Ltd., Beijing, China; QB/ABX01-009 [47]) supplied organic nitrogen and trace elements, containing ≥75% solids and ≥13% total nitrogen. All solid reagents were stored in sealed, dry containers, and batch numbers were recorded for traceability.

(2) Preparation and quality control

Each component was first dissolved separately in deionized water before mixing. Calcium chloride was added gradually with continuous stirring to control exothermic dissolution and prevent agglomeration. To minimize abiotic carbonate precipitation, NaHCO_3_-containing solutions and CaCl_2_ solutions were sterilized separately at 121 °C for 15 min, cooled to room temperature, and then mixed aseptically in the order of buffer–nutrient solution followed by calcium source addition. The initial pH was fine-tuned to 7.0 using 1.0 mol·L^−1^ HCl or NaOH. All prepared solutions were stored at 4 °C in the dark and used within one week. Before use, the pH at 25 °C and the transparency of each batch were verified, and the working solutions were used within 48 h to ensure reproducibility.

#### 2.1.5. Preparation of Soil Specimens

Previous studies reported that a 0.30% APAM content (by dry soil mass) yields optimal dry density and mechanical strength [48]. The compaction test on APAM-modified soil yielded an optimum moisture content of 13.02% and a maximum dry density of 1.97 g·cm^−3^. To ensure comparability, all test groups were compacted at a uniform moisture content of 13.02%. Six experimental groups were designed: (1) Control, containing only deionized water; (2) APAM, containing 0.30% APAM solution; and (3–6) MICP–APAM groups, containing 0.30% APAM and bacterial suspensions of OD_600_ = 0.8, 1.0, 1.2, and 1.4, respectively, with the bacterial solution replacing part of the mixing water.

The APAM dosage of 0.30% by dry soil mass was selected based on previous studies showing that this dosage range improves the dry density, interparticle bonding, and mechanical strength of fine-grained soils without causing excessive viscosity or mixing difficulty. For the tested silty clay, the APAM-modified compaction test yielded an optimum moisture content of 13.02% and a maximum dry density of 1.97 g·cm^−3^, indicating that this dosage was compatible with the compaction state used in the present specimen preparation. Therefore, 0.30% APAM was adopted as a fixed polymer content to isolate the effect of bacterial concentration in the MICP–APAM system.

For bacterial preparation, *S. pasteurii* was inoculated into the liquid culture medium and incubated at 30 °C with shaking at 200 rpm for 48 h. The OD_600_ was measured using a spectrophotometer, and the cultures were diluted to the required OD values of 0.8, 1.0, 1.2, and 1.4. Urease activity was also measured to verify metabolic stability [49]. APAM stock solution was prepared by dispersing the powder in deionized water under magnetic stirring for at least 60 min, allowed to hydrate fully, and remixed before use. The amount of water introduced by the stock solution was accounted for when calculating the total moisture content so that each group maintained a consistent total water ratio of 13.02%.

OD600 was used as an operational index to adjust the relative bacterial concentration before soil treatment. The bacterial suspension was harvested at the late exponential to early stationary phase, when urease activity was stable. Although OD600 provides a rapid and reproducible concentration-control method, it was not converted into a complete CFU-based growth curve in this study. Future work should establish OD600-CFU calibration curves and monitor the lag, exponential, stationary, and decline phases to further standardize MICP-APAM treatment.

The soil was sieved through 2.00 mm and 0.075 mm meshes. The liquid components (water, APAM solution, and bacterial suspension) were mixed with dry soil according to the target proportions and stirred manually or using a planetary mixer for 3–5 min until uniform. The mixtures were sealed in polyethylene bags and allowed to equilibrate for 24 h to achieve homogeneous water and solute distribution. Cylindrical specimens (39.1 mm diameter × 80 mm height) were then compacted in molds, demolded, and sealed with plastic film. The specimens were cured in a humidity chamber at 20 ± 1 °C and 90% relative humidity for 7 days before mechanical and physical testing.

### 2.2. Experimental Methods

#### 2.2.1. Unconfined Compressive Strength (UCS) Test

The UCS test was conducted in accordance with the Chinese standard GB/T 50123-2019 [41]. Cylindrical specimens with a diameter of 39.1 mm and a height of 80 mm were used. After curing for 7 days, the specimen surface was gently wiped to remove free water or debris and tested immediately. Loading was controlled by displacement at a rate of 1.0 mm/min until the axial stress reached its peak. The axial load *P* and axial displacement Δℎ were continuously recorded, and the axial stress was calculated using the corrected-area method. Each test group included at least three parallel specimens. If the coefficient of variation exceeded 10%, outliers were excluded and additional specimens were tested to ensure statistical consistency. To minimize end friction, a thin polytetrafluoroethylene (PTFE) sheet was placed between the specimen and the loading platens, and the flatness deviation of the end surfaces was maintained within 0.02 mm.

#### 2.2.2. Triaxial Compression Test

Unconsolidated–undrained (UU) triaxial compression tests were performed using a TSZ fully automatic triaxial testing system. The specimen geometry and preparation procedure were consistent with those used in the UCS tests. Porous stones were placed at both ends of the specimen, which was enclosed in a latex membrane. All drainage valves were kept closed, and no back pressure or consolidation was applied. Tests were conducted at a temperature of 20 ± 2 °C under confining pressures of 100, 200, and 300 kPa. Three parallel specimens were tested at each confining pressure. After the confining pressure was applied, specimens were allowed to stabilize for 5 min. Loading was displacement-controlled at an axial strain rate of 0.8% per minute [50]. Each test was terminated at the peak stress, and stress–strain curves were plotted to analyze the peak strength, yield behavior, and post-peak softening characteristics. The average response of three specimens was reported for each confining level.

#### 2.2.3. Ultrasonic Pulse Velocity (UPV) Test

A non-metallic ultrasonic testing system was used to measure the longitudinal wave velocity of the specimens. The transducers had a central frequency of approximately 50 kHz, and the measurement configuration followed a direct transmission path. Transmitting and receiving probes were positioned along the specimen axis, and a gel-based coupling agent was applied to ensure proper acoustic contact. The probes were pressed gently against the specimen until stable readings were obtained. The device was calibrated daily using a standard reference block or a specimen of known thickness and travel time. The travel time *t* (s) and the propagation path length *L* (m) were recorded, and the ultrasonic velocity was calculated as

$$V=\frac{L}{t}\;$$

$$\begin{array}{c} \mathrm{The}\;\mathrm{velocity}\;\mathrm{retention}\;\mathrm{ratio}\;\mathrm{after}\;n\;\mathrm{freeze–thaw}\;\mathrm{cycles}\;\mathrm{was}\;\mathrm{calculated}\;\mathrm{as}\\ R_{V}=\frac{V_{n}}{V_{0}} \end{array}$$

#### 2.2.4. Freeze–Thaw Cyclic Test

After 7 days of curing, specimens were subjected to air-based freeze–thaw cycling in a controlled temperature–humidity chamber. The temperature alternated between −20 °C and 20 °C, with each full cycle lasting 24 h (12 h at each temperature stage). The heating and cooling rates were controlled within 1–2 °C/min. To reduce water loss, all specimens were wrapped with plastic film during the cycles. The initial mass of each specimen was measured as *m*_0_, and the mass after *n* freeze–thaw cycles was measured as *m*_*n*_. The mass loss ratio *M*_*L*_ was calculated using

$$M_{L}=\frac{m_{0}-m_{n}}{m_{0}}\times 100\%\;$$

Mechanical and ultrasonic tests were conducted after 0, 1, 3, 6, and 9 freeze–thaw cycles. Before each test, specimens were equilibrated at room temperature for 2 h to ensure uniform thermal conditions. Any specimens with damaged wrapping or visible cracks not caused by testing were excluded from data analysis.

The nine-cycle freeze–thaw protocol was used as a controlled laboratory procedure to compare the relative degradation resistance of untreated, APAM-treated, and MICP-APAM-treated specimens. This cycle number does not represent the full service life of cold-region earthworks, where soils may experience many more cycles together with variable water supply, stress state, drainage, and temperature gradients. Therefore, the present results should be interpreted as short-term comparative durability evidence. Longer-term cyclic exposure and field-scale validation are required to evaluate service-life performance.

#### 2.2.5. Microstructural Characterization

Samples were oven-dried at 40 °C to constant mass and prepared for different analytical techniques. Powder samples were used for XRD, Fourier transform infrared spectroscopy (FTIR), and thermogravimetric–derivative thermogravimetric (TG/DTG) analyses. All powder samples were ground to pass through a 75 μm sieve. For XRD, powder was evenly spread on the sample holder and leveled to avoid orientation effects. XRD analysis employed Cu Kα radiation (λ = 1.5406 Å) at 40 kV and 30 mA, scanning from 5° to 80° (2θ) with a step size of 0.02° and a rate of 8°/min to identify calcite, aragonite, and vaterite phases and to semi-quantitatively estimate their relative contents. FTIR spectra were obtained in attenuated total reflectance (ATR) mode using finely ground powder evenly pressed against the crystal surface. The wavenumber range was 4000–400 cm^−1^, with a spectral resolution of 4 cm^−1^ and 64 scans each for background and sample. All spectra were baseline-corrected and normalized to identify characteristic vibrations of carboxylate, carbonate, and polymer functional groups. TG/DTG analysis was performed on approximately 10 ± 2 mg of powder using alumina crucibles under a nitrogen atmosphere (50 mL·min^−1^). The temperature increased from 25 °C to 1000 °C at a heating rate of 20 °C·min^−1^. The resulting weight-loss data were used to differentiate evaporation of free and bound water and to identify carbonate decomposition for estimating CaCO_3_ content. For scanning electron microscopy (SEM), small plate-like fragments approximately 5 × 5 × 2 mm were taken from fresh fracture surfaces. Dust particles were gently removed using a soft blade, and specimens were mounted on aluminum stubs with conductive adhesive. The surfaces were sputter-coated with Au to a thickness of 5–8 nm. Imaging was performed using an accelerating voltage of 5–20 kV and a working distance of 8–10 mm. Micrographs were collected at identical magnifications and imaging conditions to examine crystal morphology, cementation bridges, and pore refinement characteristics.

#### 2.2.6. Statistical Analysis

Quantitative UCS, UU triaxial, and UPV results were obtained from at least three parallel specimens. Results are reported as mean ± standard deviation. One-way analysis of variance (ANOVA) was used to evaluate intergroup differences, and Bonferroni post hoc tests were applied for pairwise comparisons when the ANOVA result was significant. A significance level of *p* < 0.05 was used. Numerical differences that were not statistically significant were interpreted as descriptive trends.

## 3. Results

### 3.1. UCS Results

**Figure 3:**
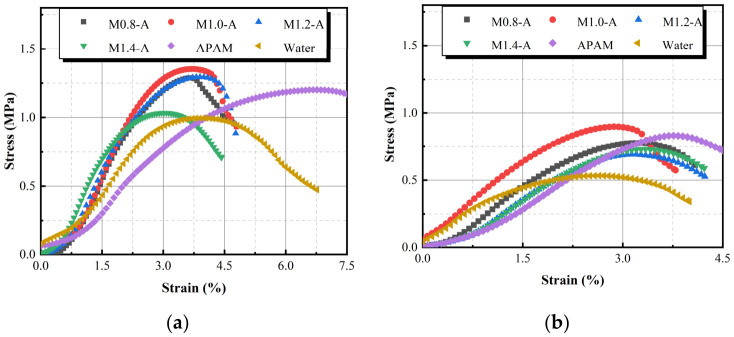
Unconfined compressive stress–strain curves after freeze–thaw cycles. (**a**) FT = 0; (**b**) FT = 9.

To evaluate the synergistic effect of APAM and MICP, UCS tests were performed on specimens cured for 7 days at 20 °C, followed by testing under 0 and 9 freeze–thaw (FT) cycles (Figure 3). As shown in Figure 3a (FT = 0), the peak strength decreased in the order of M1.0-APAM (1.35 MPa) > M1.2-APAM (1.29 MPa) > M0.8-APAM (1.28 MPa) > APAM (1.20 MPa) > M1.4-APAM (1.03 MPa) > Water (0.99 MPa). Compared with the Water group, the strength increased by approximately 36.4%, 30.3%, 29.3%, 21.2%, and 4.0%, respectively. Relative to the APAM group, variations were +12.5% (M1.0-APAM), +7.5% (M1.2-APAM), +6.7% (M0.8-APAM), and −14.2% (M1.4-APAM). All curves exhibited an initial elastic stage followed by post-peak softening. The APAM-modified specimen showed a longer ascending branch and slower post-peak reduction, indicating greater ductility and toughness [51]. Considering both strength and curve characteristics, M1.0-APAM showed the best performance under standard curing, suggesting that a moderate bacterial concentration and mineralization rate promoted the formation of a uniform and continuous CaCO_3_ cementation network [52]. Conversely, the relatively low initial strength of M1.4-APAM implied that excessive bacterial density led to crystal agglomeration, heterogeneous structure, and localized stress concentration, which reduced the load-bearing capacity [53].

After nine freeze–thaw cycles (Figure 3b), the order of peak strength changed to M1.0-APAM (0.89 MPa) > APAM (0.83 MPa) > M0.8-APAM (0.77 MPa) > M1.4-APAM (0.72 MPa) > M1.2-APAM (0.69 MPa) > Water (0.53 MPa). The strength improvements compared with the Water group were 67.9%, 56.6%, 45.3%, 35.8%, and 30.2%, respectively. Compared with the APAM group, the differences were +7.2% (M1.0-APAM), −7.2% (M0.8-APAM), −13.3% (M1.4-APAM), and −16.9% (M1.2-APAM). M1.0-APAM exhibited the highest strength retention, indicating that moderate mineralization maintained a stable cementation framework during cyclic freezing and thawing. The APAM specimen, although slightly lower in peak strength, displayed a smooth curve with a gradual post-peak decline, demonstrating excellent ductility and freeze–thaw resilience. This behavior suggests that the polymer network mitigated crack propagation and preserved structural integrity during temperature cycling [39]. In contrast, the M0.8-APAM group exhibited a significant strength reduction after freeze–thaw exposure, implying that low bacterial concentration resulted in insufficient cementation. Both M1.2-APAM and M1.4-APAM also experienced strength losses, with M1.2-APAM showing the largest decline due to excessive crystallization, pore heterogeneity, and microcrack-induced stress concentration, which weakened the overall mechanical performance [54].

Overall, both MICP and APAM treatments markedly improved the compressive performance of the silty clay. The M1.0-APAM mixture achieved a favorable balance between strength and structural stability before and after freeze–thaw cycles, representing the optimal combination. The APAM-only specimen exhibited superior ductility and freeze–thaw flexibility, effectively inhibiting crack propagation in cold-region conditions, whereas the untreated Water group showed the lowest strength and the greatest degradation after cyclic freezing and thawing.

From an engineering perspective, the post-peak response is as important as the peak strength. The APAM-containing specimens showed a smoother post-peak decline and a longer deformation stage, indicating that the polymer network improved ductility and delayed localized cracking. In the MICP–APAM composites, moderate mineralization increased stiffness and bonding, whereas excessive mineralization promoted local brittleness through heterogeneous carbonate aggregation. Therefore, M1.0-APAM represents a balance between cementation strength and deformation tolerance rather than a condition that only maximizes mineral precipitation.

### 3.2. Triaxial Shear Strength Analysis

**Figure 4:**
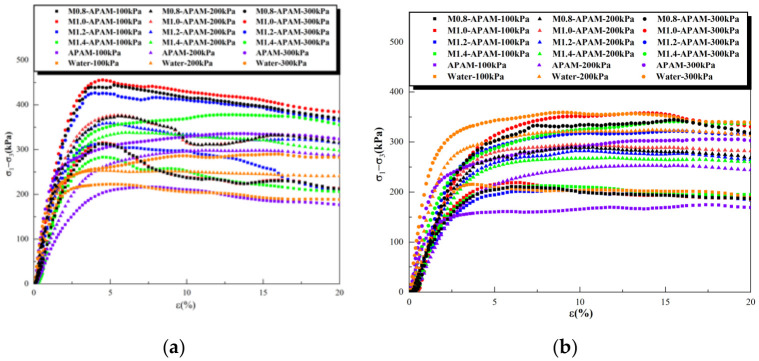
UU triaxial stress–strain curves at confining pressures of 100, 200, and 300 kPa: (**a**) FT = 0; (**b**) FT = 9.

Unconsolidated–undrained (UU) triaxial tests were performed to investigate the evolution of mechanical behavior of MICP–APAM–modified silty clay under freeze–thaw conditions. Specimens were cured at 20 °C for 7 days, then tested after 0 and 9 freeze–thaw cycles under confining pressures of 100, 200, and 300 kPa. The stress–strain responses are shown in Figure 4, where Figure 4a corresponds to FT = 0 and Figure 4b corresponds to FT = 9.

Before freeze–thaw cycling, the peak shear strength followed the order M1.0-APAM > M0.8-APAM > M1.2-APAM > APAM > M1.4-APAM > Water. This trend is consistent with the UCS results and indicates that the synergistic action of MICP and APAM can markedly enhance structural strength at an early age. M1.0-APAM achieved the highest strength at all confining pressures, suggesting that a suitable bacterial concentration promoted more uniform carbonate precipitation, and together with APAM, formed a continuous cementation network that increased interparticle cohesion and friction. M0.8-APAM ranked second. Although the amount of precipitate was lower, the structure remained relatively uniform with good interfacial bonding. For M1.2-APAM and M1.4-APAM, increasing bacterial concentration led to agglomeration and local pore clogging [55], which weakened APAM flocculation and chain-bridging effects and reduced shear strength. The APAM-only specimen relied on physical bonding within the polymer network and therefore showed limited strength enhancement. The Water specimen lacked any cementing phase and exhibited the lowest strength, depending mainly on frictional resistance mobilized under confinement.

After nine freeze–thaw cycles, the ranking changed to M1.0-APAM > Water > M0.8-APAM > M1.4-APAM > M1.2-APAM > APAM. All groups experienced strength reduction, but the extent of degradation differed. M1.0-APAM still showed the highest strength, indicating that the carbonate cementation framework remained effective under cyclic thermal loading. M0.8-APAM exhibited a smaller decline, implying that lower bacterial concentration provided greater structural resilience during freeze–thaw. Although M1.4-APAM had a looser initial structure, the presence of fine crystals together with residual APAM support produced slightly higher strength than M1.2-APAM. The M1.2-APAM specimens suffered the most severe deterioration, which is attributed to crystal spalling, pore enlargement, and microcrack coalescence during cycling. The Water specimens displayed a relative increase in peak strength compared with their FT = 0 state. The longer rising branch and higher residual strength under all confining pressures suggest particle rearrangement and pore compaction during cycling, which produced a self-densified structure with more uniform pore-water pressure and higher effective stress [56]. This improvement reflects physical densification rather than enhanced cementation and may not be sustainable over longer service periods.

The APAM-only group showed a pronounced strength decrease and more evident post-peak softening. This behavior can be explained by polymer chain softening and reduced interfacial bonding under cyclic freezing and thawing, which progressively undermined particle bridging. Prior studies reported that APAM chains undergo mechanical fatigue and partial scission during freeze–thaw, creating weak bands that slide along particle interfaces and initiate localized shear failure [39]. In summary, both MICP and APAM enhance shear resistance before freeze–thaw, while their durability differs under cyclic conditions. The MICP–APAM composite at an appropriate bacterial concentration, exemplified by OD600 = 1.0, provides the best balance between strength level and stability and maintains a higher load-bearing capacity after cycling. APAM alone improves deformability but is susceptible to freeze–thaw degradation. The Water group may show short-term densification under confinement but lacks persistent cementation and therefore offers limited long-term benefit.

### 3.3. UPV Analysis

**Figure 5:**
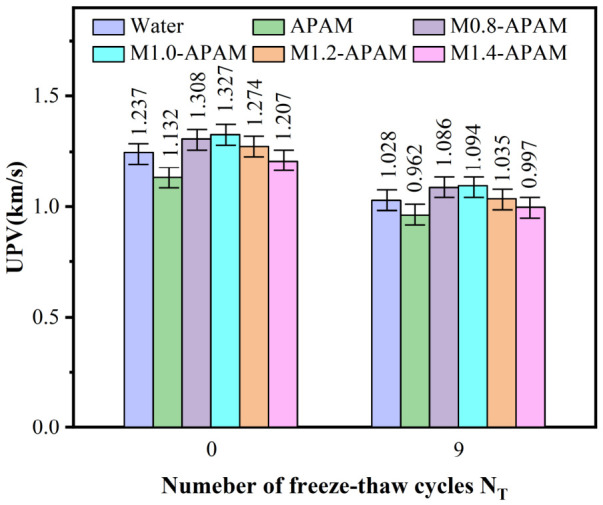
UPV of specimens before and after freeze–thaw cycles (FT = 0 and 9).

Figure 5 shows the UPV of specimens before (FT = 0) and after freeze–thaw cycles (FT = 9). At FT = 0, the UPV values of each group were as follows: Water 1.237 km/s, APAM 1.132 km/s, M0.8-APAM 1.308 km/s, M1.0-APAM 1.327 km/s, M1.2-APAM 1.274 km/s, and M1.4-APAM 1.207 km/s. The velocity sequence can be expressed as M1.0-APAM > M0.8-APAM > M1.2-APAM > Water > M1.4-APAM > APAM. The relatively high velocities of M1.0-APAM and M0.8-APAM indicate that these specimens developed denser microstructures and more continuous interfaces between particles and cemented phases in the initial state. The slightly lower value of M1.2-APAM suggests partial agglomeration or localized pore blockage that limited wave propagation, while the further reduction in M1.4-APAM reflects increased structural heterogeneity and stronger scattering effects. The APAM group exhibited the lowest UPV, attributed to the low acoustic impedance of the flexible polymer phase compared with the mineral skeleton. The soft polymer bridges encapsulating the soil particles increased interfacial scattering and energy dissipation, thereby reducing the effective propagation velocity.

After nine freeze–thaw cycles, the UPV values were as follows: Water 1.028 km/s, APAM 0.962 km/s, M0.8-APAM 1.086 km/s, M1.0-APAM 1.104 km/s, M1.2-APAM 1.035 km/s, and M1.4-APAM 0.997 km/s. The corresponding velocity retention ratios *Rv* = *V*9/*V*0 were approximately 0.85 for APAM, 0.83 for M1.0-APAM, 0.83 for M0.8-APAM, 0.83 for Water, 0.83 for M1.4-APAM, and 0.81 for M1.2-APAM. The overall decrease in velocity indicates that freeze–thaw cycles induced the formation of microcracks and increased pore connectivity in all specimens. Among them, APAM exhibited the highest retention ratio, demonstrating that its water-retention and viscous-regulation capacity partially inhibited crack propagation during cycling. However, its absolute UPV remained lower than those of the mineralized groups, reflecting the soft and ductile nature of the polymer matrix. Both M1.0-APAM and M0.8-APAM maintained higher absolute velocities and retention ratios, suggesting that moderate mineralization density and continuous interfaces help preserve compactness after freeze–thaw cycles. M1.2-APAM showed the lowest retention ratio, consistent with the UCS and triaxial results, which can be attributed to non-uniform crystallization that produced localized stress concentration and microcrack coalescence. The lower absolute velocity and moderate retention of M1.4-APAM indicate that the fine and unevenly distributed crystals generated at high bacterial concentrations increased scattering and energy attenuation. The Water group exhibited a significant velocity decline after freeze–thaw cycles, reflecting the development of microcracks and enhanced pore connectivity in the untreated soil.

The apparent increase in the Water-group triaxial strength after freeze–thaw cycling should be interpreted with caution. Under UU triaxial loading, confining pressure can promote particle rearrangement and temporary pore collapse, leading to a denser stress-bearing skeleton and higher short-term peak strength. However, this confinement-induced densification does not indicate improved internal integrity. The corresponding UPV decrease shows that freeze–thaw cycling increased microcrack density and pore connectivity, which caused stronger wave scattering and lower ultrasonic velocity. Thus, the Water group exhibited short-term mechanical densification under confinement but still suffered internal freeze–thaw damage.

Overall, the UPV results are consistent with the UCS and triaxial test outcomes. M1.0-APAM exhibited the highest compactness and stability before and after freeze–thaw cycles, while APAM demonstrated greater resistance to crack propagation rather than higher stiffness. In contrast, specimens with high bacterial concentration exhibited structural heterogeneity and enhanced scattering, which were detrimental to both acoustic transmission and mechanical performance.

### 3.4. SEM Analysis

#### 3.4.1. SEM Observations Before Freeze–Thaw Cycles

**Figure 6:**
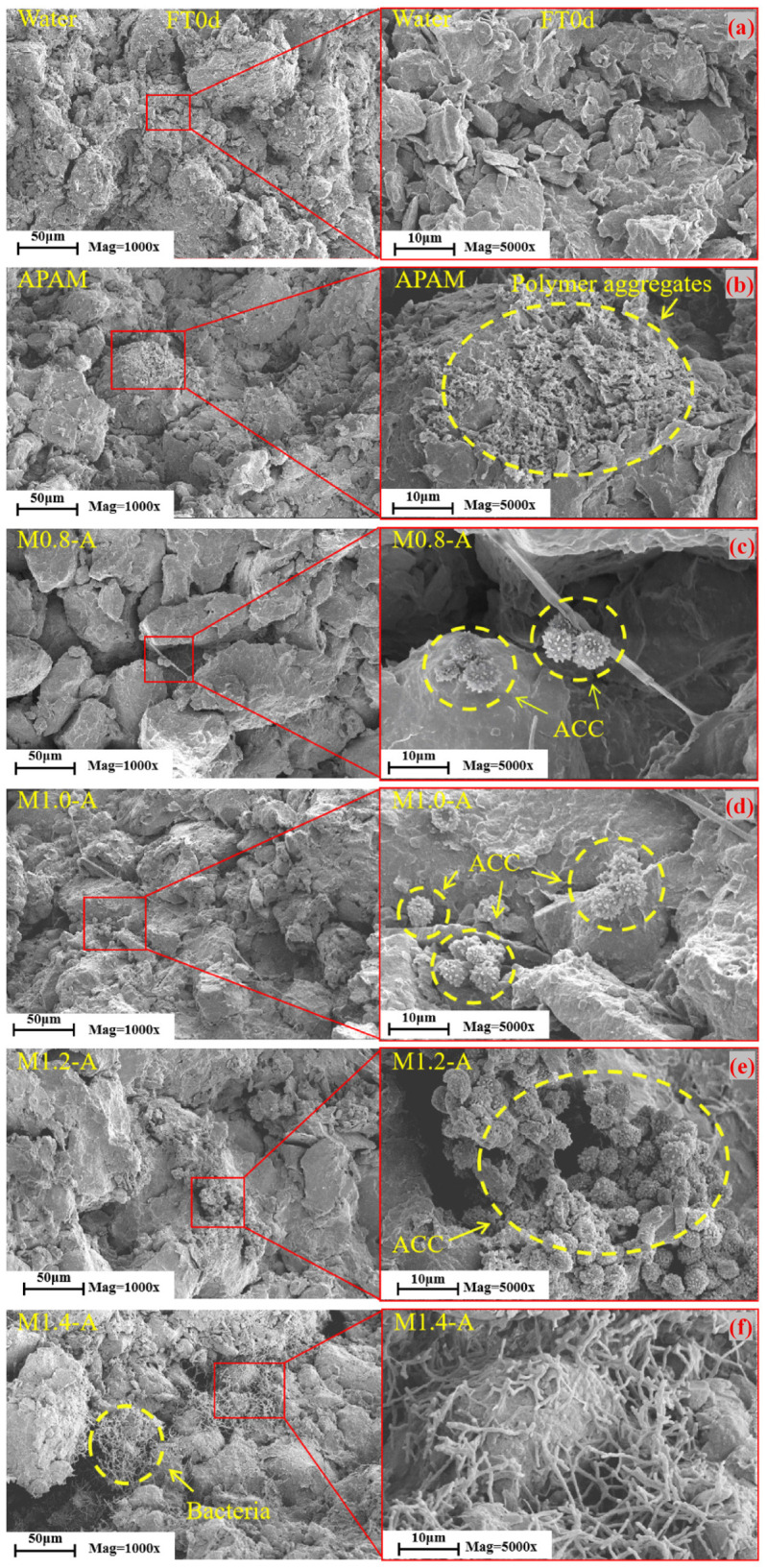
SEM images of samples before freeze–thaw cycling (FT = 0). (**a**) Water; (**b**) APAM; (**c**) M0.8-APAM; (**d**) M1.0-APAM; (**e**) M1.2-APAM; (**f**) M1.4-APAM.

Figure 6 presents the microstructures of the differently treated specimens prior to freeze–thaw cycling. In the control (Figure 6a), the Water group shows a loose, platy aggregate with clear particle boundaries and a high pore fraction, and interparticle stability is provided mainly by frictional contacts with no discernible cementing phase. In the APAM group (Figure 6b), distinct polymer aggregates are observed. The flexible film-like polymer bridges and partially coats adjacent grains, resulting in narrowed pore throats and locally filled voids. The structure is tighter than the control, yet the absence of an inorganic cement limits macroscopic strength enhancement.

MICP-treated specimens (Figure 6c–e) exhibited the characteristic coexistence of filaments and clustered deposits. The clusters were commonly raspberry-like, with typical sizes of approximately 1–3 μm, and were interpreted as carbonate-like precipitates that may include amorphous or microcrystalline CaCO_3_. In M0.8-APAM, clusters were sparse and filaments were discontinuous, indicating limited nucleation sites and an incomplete cementation network. In M1.0-APAM, small clusters and thin films were uniformly distributed and locally coalesced into continuous surface coverage along grain edges and pore throats, producing pore refinement and a more homogeneous cemented fabric that represents the most favorable microstructural state in this series. In M1.2-APAM, the cluster density increased and stacked aggregates formed, generating strong local filling but reduced spatial uniformity and a higher risk of stress concentration.

ACC refers to amorphous calcium carbonate, a transient carbonate phase that may form during biologically induced mineralization. In this study, SEM morphology was used to observe particle bonding, pore filling, and deposit distribution, but morphology alone cannot conclusively distinguish bacterial cells, ACC, crystalline carbonate, and polymer residues. Therefore, the SEM annotations are interpreted as morphology-based indicators, and the interpretation is supported by TG/DTG carbonate-decomposition behavior, XRD calcite reflections, and FTIR carbonate-related bands. Definitive phase assignment would require EDS mapping, point analysis, or complementary high-resolution characterization.

At the highest bacterial concentration, M1.4-APAM (Figure 6f) transitions toward a mesh-like and nodular three-dimensional skeleton. Slender linear deposits and biofilm-related strands are evident. Although this framework creates pervasive bridging and partitions large voids into smaller pores, the enclosed microcavities and elongated strands are prone to local buckling and interfacial instability under axial loading, which lowers the load-bearing efficiency. At the macroscopic level, this behavior is consistent with the drop in UCS from 1.35 MPa to 1.03 MPa, reflecting the adverse effects of non-uniform deposition and interfacial stress concentration at excessive inoculation.

Overall, the micrographs indicate that the modification efficacy is governed by the interplay between MICP nucleation and APAM bridging. When OD600 is approximately 1.0, nucleation and growth are balanced with diffusion, yielding a continuous but not overly thick film-cluster composite that reconciles compactness with toughness. At OD600 = 0.8, coverage is insufficient due to limited nucleation. At OD600 ≥ 1.2, aggregation becomes pronounced and spatial uniformity decreases. The raspberry-like carbonate clusters are most evident in the OD600 = 0.8–1.2 range and are most uniformly distributed in M1.0-APAM, which aligns with the peak strength measured at the macroscale.

#### 3.4.2. SEM Observations After 9 Freeze–Thaw Cycles

**Figure 7:**
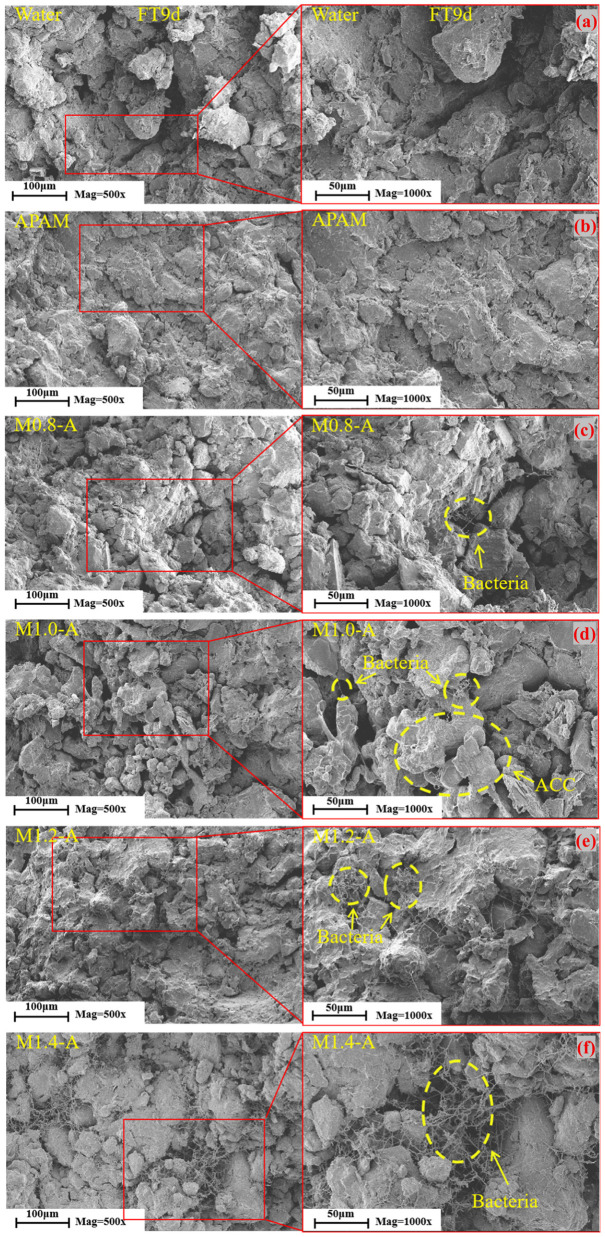
SEM images of samples after 9 freeze–thaw cycles. (**a**) Water; (**b**) APAM; (**c**) M0.8-APAM; (**d**) M1.0-APAM; (**e**) M1.2-APAM; (**f**) M1.4-APAM.

Figure 7 illustrates the microstructural morphology of the specimens after nine freeze–thaw cycles. The Water specimen (Figure 7a) exhibited loosely stacked platy particles with open pores, showing clear evidence of particle rearrangement and local detachment induced by freeze–thaw action [57]. The APAM specimen (Figure 7b) displayed discontinuous thin films and polymer aggregates. Small cracks were observed on the film surfaces, indicating that the polymer network softened and contracted during cyclic freezing and thawing, leading to reduced bridging capability and the absence of inorganic cementation support [58].

In the M0.8-APAM sample (Figure 7c), only a few scattered bridges and filamentous biological remnants were visible. The pore refinement was discontinuous, consistent with the limited nucleation expected at low bacterial concentrations. The M1.0-APAM sample (Figure 7d) showed relatively uniform spherical clusters and thin-film composite cementation. The raspberry-like carbonate clusters were distributed along grain contacts and pore throats, forming a continuous yet moderate coverage that preserved the integrity of the cementation network after cyclic freezing and thawing [59,60]. This morphology corresponded well with its high strength and ultrasonic velocity retention. In contrast, the M1.2-APAM sample (Figure 7e) exhibited a dense accumulation of clusters and plate-like deposits with reduced spatial uniformity and irregular pores, indicating diffusion limitation and increased stress-concentration potential, which matched its greater strength and UPV degradation [61].

At the highest inoculation level, M1.4-APAM (Figure 7f) was dominated by a mesh-like and nodular skeleton structure, where numerous microcavities and elongated filaments remained. These features suggest that the microstructure is prone to local buckling and interfacial instability under load, reducing its load-bearing efficiency. Overall, moderate bacterial concentration (OD600 ≈ 1.0) favored a well-balanced microstructure characterized by uniform coverage, adequate filling, and stable bridging. Low concentrations led to insufficient coverage, while excessively high concentrations promoted network-like and aggregated structures that were detrimental to post-freeze–thaw mechanical performance.

The SEM images indicate that the MICP–APAM system produced a composite network consisting of APAM-associated film bridges, carbonate-like clusters, and mineral-polymer cementation layers. The APAM phase bridged adjacent particles and narrowed pore throats, while the carbonate precipitates filled voids and formed rigid contact points. This hybrid network improved particle bonding and stress transfer, reduced pore connectivity, and limited freeze–thaw-induced crack propagation. The most continuous and uniform network was observed in the M1.0-APAM group, which is consistent with its superior UCS, triaxial strength, and UPV performance.

### 3.5. TG Analysis

**Figure 8:**
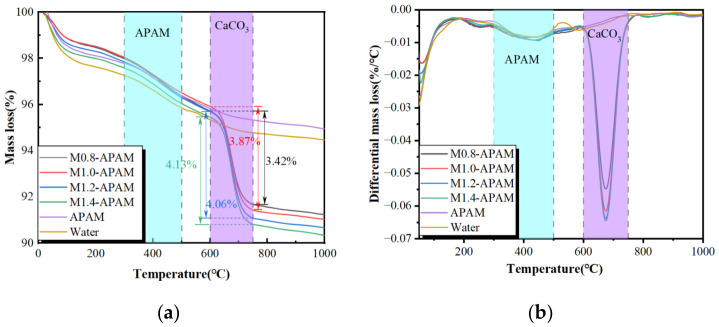
(**a**) TG and (**b**) DTG curves of samples before the freeze–thaw cycle.

Figure 8 shows the TG curves (Figure 8a) and DTG curves (Figure 8b) of samples before the freeze–thaw cycle. Two main thermal events can be identified: the first, between approximately 300–500 °C, corresponds to the thermal degradation of APAM, and the second, between 600–750 °C, corresponds to the decomposition of CaCO_3_. The gentle weight losses at lower temperatures are associated with the evaporation of free and weakly bound water and the oxidation of small amounts of residual organic matter.

In the 300–500 °C range, all samples exhibited a distinct weight loss accompanied by a corresponding DTG trough, which can be attributed to the degradation of the APAM molecular backbone (–CH_2_–CH(CONH_2_)–) and its side groups (carboxylate and amide functionalities). The measured weight losses in this interval were approximately 4.13% for M0.8-APAM, 4.06% for M1.2-APAM, 3.87% for M1.0-APAM, and 3.42% for M1.4-APAM, whereas the APAM-only and Water groups showed smaller losses. These differences indicate variations in organic-phase content and thermal stability, confirming that the organic component effectively participated in the soil modification process. During decomposition, the polymer released volatile species such as NH_3_, CO_2_, NO, and NO_2_ while forming local carbonized residues that may have altered the surface energy of soil particles and the microstructure of pore interfaces.

In the 600–750 °C range, all mineralized specimens exhibited a sharp weight-loss step and a pronounced DTG peak corresponding to the decomposition reaction of CaCO_3_ → CaO + CO_2_. The CO_2_-related weight losses (ΔmCO_2_) were approximately 4.13% for M0.8-APAM, 4.06% for M1.2-APAM, 3.87% for M1.0-APAM, and 3.42% for M1.4-APAM, while the APAM and Water groups exhibited losses of only about 1%, close to the detection limit. These results demonstrate that the MICP process substantially increased the CaCO_3_ content in the soil matrix, whereas the APAM and Water specimens contained only trace amounts of carbonates inherited from the original silty clay (mainly dolomite and related minerals) without evidence of new mineral formation.

It is worth noting two key observations. First, although M1.4-APAM and M1.2-APAM exhibited higher CaCO_3_ contents, their DTG peaks were broader and less sharp, with wider thermal-decomposition steps, suggesting crystal agglomeration, structural heterogeneity, and restricted CO_2_ diffusion. These microstructural features explain the lower UPV and more pronounced mechanical degradation observed for these groups. Second, M1.0-APAM contained slightly less CaCO_3_ than M1.2-APAM and M1.4-APAM but displayed a more concentrated DTG peak, indicating a narrower crystal size distribution and more continuous interfaces, consistent with its superior strength and stability in UCS and triaxial tests. Overall, these findings confirm that the mechanical and acoustic performance of the modified soil is jointly controlled by mineralization amount, structural uniformity, and pore connectivity, rather than by CaCO_3_ content alone.

### 3.6. XRD Analysis

**Figure 9:**
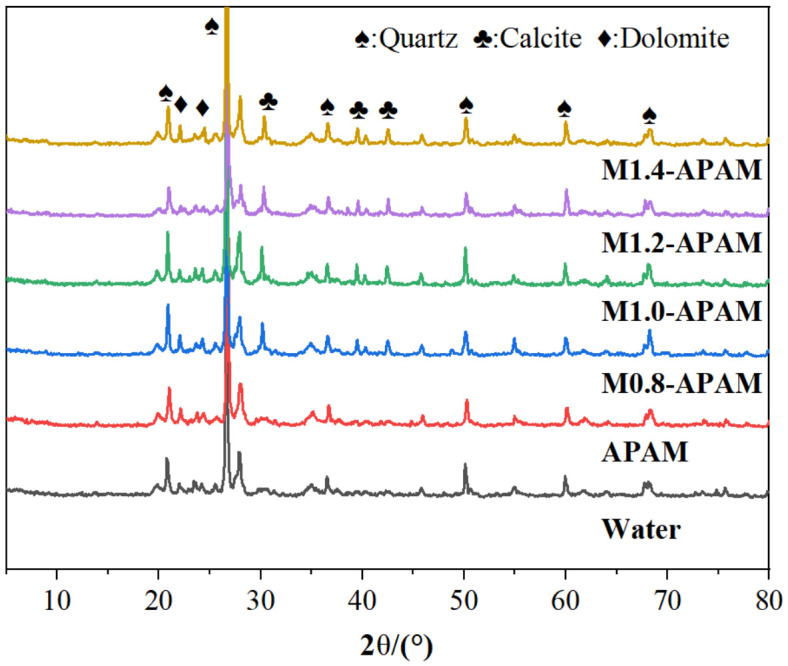
XRD results of samples before the freeze–thaw cycle.

XRD was used to identify crystalline mineral phases and assess carbonate deposition under different treatments. The principal reflections correspond to quartz, calcite, and dolomite (Figure 9). Compared with the Water and APAM groups, the MICP–APAM specimens exhibited discernible calcite peaks at approximately 2θ ≈ 29.4°, 39.4°, 43.1°, 47.5°, 56.6°, and 64.0°, indicating the presence of biologically induced calcium carbonate. The intergroup differences in calcite intensity were modest, which is consistent with limited deposition and fine crystallite size. Even so, the MICP–APAM specimens showed stronger calcite signals than the APAM and Water specimens, supporting the formation of additional CaCO_3_ in the biologically treated soils. In the APAM and Water groups, calcite signals were very weak and mainly reflect the background carbonate associated with the silty-clay matrix rather than newly precipitated phases. When combined with the TG/DTG results, which showed greater CO_2_ weight loss between 600 and 750 °C for the MICP-treated specimens, the increase in calcite peak intensity provides consistent evidence for enhanced carbonate content after microbial treatment. APAM alone does not generate new carbonate, although its polymer network can alter Ca^2+^ transport and nucleation microenvironments, which may indirectly influence the spatial distribution of mineralization.

The optimal performance of M1.0-APAM should not be interpreted as the result of maximum CaCO_3_ content alone. TG/DTG showed carbonate-related mass loss in the 600–750 °C interval for the MICP–APAM groups, and XRD confirmed calcite as the dominant carbonate phase. Although higher-OD600 groups may contain more locally accumulated carbonate, their broader DTG response and less uniform SEM morphology indicate agglomeration and spatial heterogeneity. Therefore, OD600 = 1.0 represents the best balance among carbonate generation, deposition uniformity, pore refinement, and APAM-mediated particle bonding.

### 3.7. FTIR Analysis

**Figure 10:**
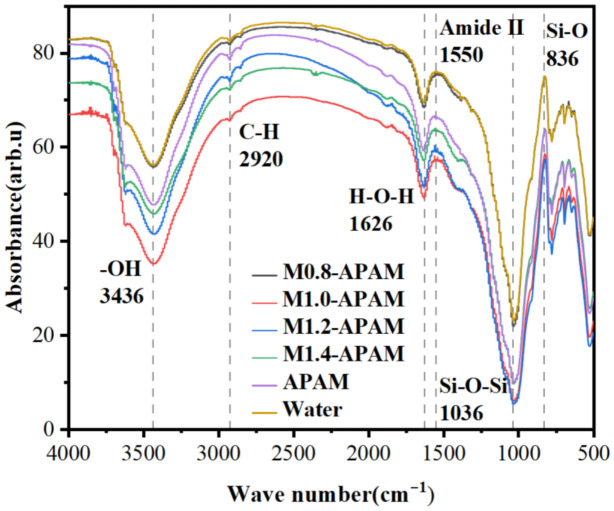
FTIR results of samples before the freeze–thaw cycle.

Figure 10 shows prominent absorption bands at 3436, 2920, 1626, 1550, 1036, and 836 cm^−1^. The broad band at 3436 cm^−1^ is assigned to stretching vibrations of −OH with a minor contribution from −NH. Its slightly higher intensity in the MICP–APAM groups indicates strengthened hydrogen bonding, which is consistent with water-retentive complexes formed by the polymer and carbonate products. The weak band near 2920 cm^−1^ corresponds to C–H stretching and is more evident in the APAM-containing specimens, reflecting the presence of the organic phase. The band at 1626 cm^−1^ arises from H–O–H bending. Its reduced intensity in the composite groups suggests a lower fraction of bound water and partial pore filling. The 1550 cm^−1^ band is the characteristic amide II vibration that combines N–H bending with C–N stretching. It is strongest in the APAM group and gradually weakens when MICP participates, indicating coordination or surface adsorption of amide groups with Ca^2+^ or carbonate products [62]. The Si–O–Si symmetric stretching at 1036 cm^−1^ and the Si–O bending near 836 cm^−1^ show nearly identical positions and intensities among all groups, implying that the silicate skeleton remains intact. Carbonate-related features appear only as weak shoulders or inflections in the 870–900 cm^−1^ and 700–730 cm^−1^ regions, which can be attributed to the ν_2_ and ν_4_ modes of CO_3_^2−^. The ν_3_ band in the 1415–1470 cm^−1^ range is not clearly resolved because it is overlapped by the strong amide II side band and broad mineral background absorptions. This masking effect arises from the inherently stronger absorptions of APAM and silicate frameworks relative to carbonate bands, so CaCO_3_ is reflected as weak features in FTIR. Taken together with the calcite reflection at 2θ ≈ 29.4° in XRD and the CO_2_ weight loss between 600 and 750 °C in TG/DTG, the FTIR results provide qualitative support for the presence of MICP-derived CaCO_3_ while highlighting the spectral limitations of carbonate identification in polymer–silicate matrices.

## 4. Discussion

The results indicate that the synergistic modification of silty clay using MICP and APAM effectively improves the soil structure, strength, and freeze–thaw durability. The mechanical performance is jointly governed by the amount of mineralization, the uniformity of the deposition, and the connectivity of the pores rather than by the total CaCO_3_ content alone. At a bacterial concentration of OD600 around 1.0, the rates of nucleation and ion diffusion are balanced, forming uniformly distributed carbonate clusters that bridge adjacent particles and create a continuous and flexible cementation network. The polymer chains facilitate this process by regulating moisture and ion transport and by providing viscous and hydrogen-bonding control, which helps reduce stress concentration and restrain water migration during freezing. Consequently, the specimens with moderate bacterial concentration maintain the highest strength and ultrasonic velocity after multiple freeze–thaw cycles. When the bacterial concentration is too high, excessive precipitation and crystal agglomeration result in structural nonuniformity and localized weakness, whereas at low concentrations, the limited nucleation and incomplete cementation lead to insufficient bonding. The TG/DTG, XRD, FTIR, and SEM results collectively confirm that the MICP–APAM composite promotes fine, well-dispersed calcite formation within the polymer matrix, producing a dense but ductile structure that enhances both stiffness and crack resistance. This cooperative mechanism between the inorganic and organic phases allows the material to maintain strength and integrity under freeze–thaw conditions and provides a mechanistic basis for the development of durable and environmentally compatible stabilizers for cold-region soils.

Compared with previously reported MICP-treated soils, the present MICP–APAM system shows that the optimum bacterial level is governed not only by carbonate yield, but also by the uniformity of deposition within a polymer-regulated pore network. Earlier MICP studies reported that excessive bacterial density can cause rapid local precipitation and pore clogging, while polymer-assisted treatments can improve water retention, particle bridging, and ductility. The present results are consistent with these findings: OD600 = 1.0 produced the best balance between strength enhancement, UPV retention, and uniform cluster–film microstructure, whereas higher OD600 levels promoted agglomeration and weaker post-freeze–thaw performance.

A conventional cement- or lime-stabilized positive-control group was not included in the present experimental design. Therefore, the present comparison focuses on untreated soil, APAM-treated soil, and MICP–APAM-treated soil under identical laboratory conditions. To provide an engineering benchmark, future studies should include cement-treated and lime-treated groups and compare their strength, freeze–thaw durability, environmental impact, and treatment cost with those of the MICP–APAM approach.

For field implementation, the MICP–APAM treatment would require scalable bacterial cultivation, uniform distribution of bacterial suspension and cementation solution, control of APAM dosage and viscosity, and curing conditions that maintain adequate moisture and temperature. In cold-region earthworks, seasonal temperature fluctuation, drainage condition, soil heterogeneity, and repeated traffic or slope loading may influence mineralization efficiency and polymer stability. APAM is widely used as a soil conditioner and flocculant, but its dosage, residual monomer content, migration behavior, and long-term environmental safety should be evaluated for each project. Compared with cement stabilization, the MICP–APAM approach may reduce chemical input and improve environmental compatibility, but its cost, curing time, microbial activity control, and construction standardization require further pilot-scale verification.

The present study also has several limitations. First, the APAM content was fixed at 0.30%, and the interaction between APAM dosage, molecular weight, bacterial concentration, and soil type requires further optimization. Second, the SEM-based interpretation mainly reflects local fracture-surface morphology; more systematic pore-size distribution analysis, crack-density measurement, carbonate-coverage mapping, and micro-CT observations are needed to obtain three-dimensional quantitative evidence. Third, the laboratory freeze–thaw protocol provides short-term comparative durability evidence, while long-term cyclic exposure and field-scale verification remain necessary before engineering application.

## 5. Conclusions

This study explored the synergistic modification mechanism of silty clay using MICP and APAM under freeze–thaw conditions. The influence of bacterial concentration on mechanical behavior, freeze–thaw durability, and microstructural evolution was systematically evaluated, and the coupled strengthening mechanism was elucidated through multi-scale experimental characterization. The main conclusions are as follows:

(1) The UCS and UU tests showed that MICP–APAM modification markedly enhanced the strength and stability of silty clay. All modified specimens exhibited significantly higher strength than the untreated soil. The strength followed a single-peak trend with increasing bacterial concentration, and the OD600 = 1.0 group achieved the optimal balance of stiffness and ductility. The initial UCS and post-freeze–thaw UCS of this group reached 1.35 MPa and 0.89 MPa, respectively, indicating strong structural integrity and superior resistance to freeze–thaw degradation.
(2) UPV results demonstrated that the MICP–APAM composites had improved compactness and reduced internal defects compared with the control. The M1.0-APAM and M0.8-APAM groups maintained the highest ultrasonic velocities before and after freeze–thaw cycling, confirming that moderate mineralization density and polymer bridging effectively preserved pore continuity and suppressed microcrack propagation.
(3) TG/DTG and XRD analyses confirmed calcite as the major carbonate phase, and the 600–750 °C decomposition interval corresponded to CO_2_ release from CaCO_3_. The DTG peak of the M1.0-APAM group was narrower and more concentrated, indicating homogeneous mineralization and stable interfaces. FTIR spectra revealed weak carbonate absorption due to overlap with strong polymer and silicate bands, yet provided qualitative evidence of CaCO_3_ formation. These findings demonstrated that strength improvement was governed by the combined effects of mineralization degree, structural uniformity, and pore connectivity rather than by carbonate content alone.
(4) SEM observations showed that the MICP–APAM composite effectively reconstructed the soil microstructure. The OD600 = 1.0 specimen formed a uniform network of fine spherical clusters and thin-film cementation layers, providing continuous particle bonding and enhanced pore filling. Lower bacterial concentration resulted in insufficient nucleation, while higher concentration caused crystal agglomeration and spatial heterogeneity, both leading to inferior mechanical performance.

In summary, the synergy between APAM and MICP achieved a stable balance between mineralization and flexibility, effectively improving the strength and freeze–thaw durability of silty clay. The study provides a theoretical basis and practical reference for developing sustainable and durable soil-stabilization technologies in seasonally frozen regions.

The present results demonstrate the short-term comparative freeze–thaw resistance of the MICP–APAM system under controlled laboratory conditions. The OD600 = 1.0 group showed the best overall performance because it achieved a balanced combination of carbonate formation, deposition uniformity, pore refinement, and polymer-mediated particle bonding, rather than simply producing the largest carbonate amount. Long-term cyclic exposure, benchmark comparison with conventional stabilizers, and field-scale verification remain necessary before large-scale engineering application.

## Figures and Tables

**Table 1:** Basic physical properties of the test soil.

Property	Unit	Value
Specific gravity	-	2.72
Plastic limit	%	22.5
Liquid limit	%	34.6
Plasticity index	-	12.1
Optimal moisture content (OMC)	%	14.0
Maximum dry density (MDD)	g·cm^−3^	1.87

## Data Availability

The original contributions presented in this study are included in the article. Further inquiries can be directed to the corresponding author.

## Abbreviations

The following abbreviations are used in this manuscript:

MICP	Microbially induced carbonate precipitation
APAM	Anionic polyacrylamide
PAM	Polyacrylamide
EICP	Enzyme-induced carbonate precipitation
MIPP	Microbially induced phosphate precipitation
UCS	Unconfined compressive strength
UU	Unconsolidated–undrained
UPV	Ultrasonic pulse velocity
FT	Freeze–thaw
SEM	Scanning electron microscopy
TG	Thermogravimetry
DTG	Derivative thermogravimetry
XRD	X-ray diffraction
FTIR	Fourier transform infrared spectroscopy
OD_600_	Optical density at 600 nm
OMC	Optimum moisture content
MDD	Maximum dry density
ATR	Attenuated total reflectance
PTFE	Polytetrafluoroethylene
